# Cytotoxicity and Oral Acute Toxicity Studies of *Lantana camara* Leaf Extract

**DOI:** 10.3390/molecules16053663

**Published:** 2011-05-03

**Authors:** Badakhshan Mahdi Pour, Lachimanan Yoga Latha, Sreenivasan Sasidharan

**Affiliations:** 1 Department of Biotechnology, Faculty of Applied Sciences, AIMST University, Jalan Bedong-Semeling, Batu 3 ½, Bukit Air Nasi, Bedong 08100, Kedah, Malaysia; 2 Institute for Research in Molecular Medicine (INFORMM), University Sains Malaysia, 11800, Pulau Pinang, Malaysia

**Keywords:** *Lantana camara*, cytotoxicity, oral acute toxicity, Vero cell, plant extract

## Abstract

*Background***:** The objective of this study was to investigate the toxicity of *Lantana camara* methanol extract. *Methods***:** In order to evaluate the toxicity of *Lantana camara*, the acute toxicity of the methanolic extract on adult mice and cytotoxicity test on Vero cell line were investigated. A fixed large dose of 2 g/kg body weight of *L. camara* leaf extract was administrated by a single oral gavage according to the OECD procedure. *Results***:** In 2 weeks, *L. camara* leaf extract showed no obvious acute toxicity. While female mice lost body weight after being treated with single dose of leaf extract in acute toxicity test, male ones lost organ mass, particularly for heart and kidney. The biochemical liver function tests showed significantly elevated TBIL and ALT in the *L. camara* leaf extract treated female mice group compared with the control group. Cytotoxicity effect of leaf extract of *L. camara* was estimated through a MTT assay. Cytotoxicity tests on Vero cell line disclosed that leaf extract at concentrations up to 500 µg/mL inhibited the growth of cells 2.5 times less than did Triton 100× 1%. More interestingly, the cytotoxicity initiated to decline at elevated concentrations of this extract. *Conclusions***:** The results of both tests confirm that *L. camara* shows a pro toxic effect.

## 1. Introduction

Herbs have recently attracted attention as health beneficial foods and as source materials for drug development. They offer a potential natural health care approach that focuses on protecting and restoring health. Toxicity screening models provide important preliminary data to help select natural remedies with potential health beneficial properties for future work. As part of a permanent screening program searching for natural products with beneficial biological activity properties, the current investigation reports the toxicity of *Lantana camara * L. (Verbenaceae) methanol extract. 

Folk healers in Asia and South America have used *Lantana* species, including *L. camara*, for centuries to treat various human ailments such as dermatological and gastrointestinal diseases, tetanus, malaria and tumors [1]. Different parts of *L. camara* are used for the treatment of various human ailments such as itches, cuts, ulcers, swellings, bilious fever, catarrh, eczema, tetanus, malaria, tumors and rheumatism [2,3]. In the Kancheepuram district of Tamil Nadu, a handful of flowers are grounded with coconut oil and applied topically on the head to get relief from headache [4,5]. *L. camara* is one of the most prevalent and noxious weeds, causing hepatotoxicity in grazing animals [6]. On the contrary, *L. camara* has been reported to possess a number of medicinal properties to treat various human ailments such as dermatological and gastrointestinal diseases, tetanus, malaria and tumors [7,8]. Some metabolites isolated from their leaves possess antithrombin activity [9], antiinflammatory, antinociceptive and antipyretic activity [10,11]. Considering both the ethnobotanical and pharmacological applications of the plant, the aim of this study was to investigate the possible toxic effects of the leaf extract of *L. camara* in mice and cytotoxicity tests.

## 2. Results and Discussion

### 2.1. Acute Oral Toxicity Study

**Figure 1:**
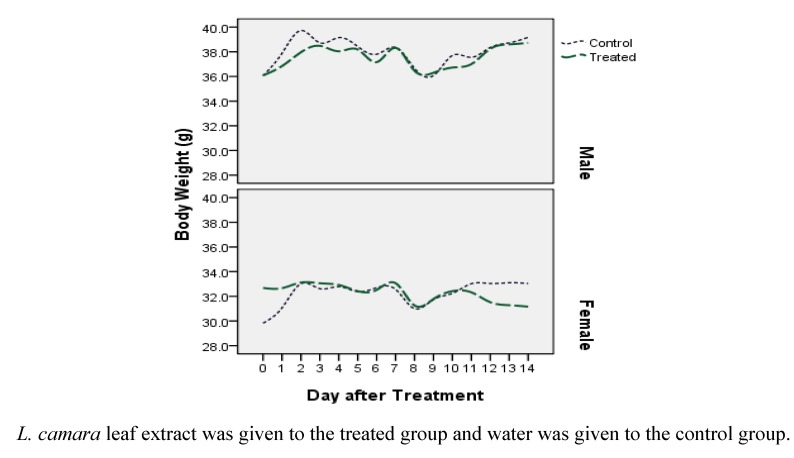
Comparing the changes in body mass across sexes in control and treatment groups during fortnight observation based on acute toxicity test of leaf extract of *L. camara* on Swiss Albino Mice.

To establish the safety of the extract, 2,000 mg/kg methanol extract was administered to both male and female mice. We observed no significant toxic signs or death during the 14 day observation period. None of the mice showed clinical toxic signs such as anorexia, depression, lethargy, jaundice, dermatitis and also, no mortality happened throughout the examination. Nevertheless, Figure 1 suggests that based on differential analysis on body weight, female mice are more sensitive to the leaf extract than male ones. While mean body weight for females in the treated group was 32.68 ± 2.74 g at the beginning of study, it decreased erratically to 31.16 ± 1.74 g at the end. Spearman's rho nonparametric test confirms that this reduction is significant (correlation coefficient = -0.176, *P* < 0.01). For three other groups, the body weight after the treated group is more than that at the control groups. Since the control and treated males profile (Figure 1) are comparable, it can be concluded that leaf extract of *L. camara* doesn’t influence the body weight of male Swiss albino mice. On the other hand, divergent profile especially at extremes of control and treated females (Figure 1) demonstrate the possibility of short term and long term toxicity in this sex.

Johnson and Jensen [12] also found that female red kangaroo is more vulnerable to *L. camara* than the male animal and even the yearling. On the basis of Sharma *et al.* [13] study, the decline in body mass could be preliminarily attributed to a decrease in food intake which is related to the release and absorption of toxins in the gastrointestinal tract.

The fluctuations in the curves (Figure 1) do not reflex toxicity-related effects, but rather the impact of variations in diet. As the food for all mice changed in days between 7 and 10, there are dramatic reductions in body weight among treated as well as control groups in both sexes. The sharp increase in body mass during the first two days of investigation may also hint that the pervious diet included lesser amounts of carbohydrates or lipids.

**Figure 2:**
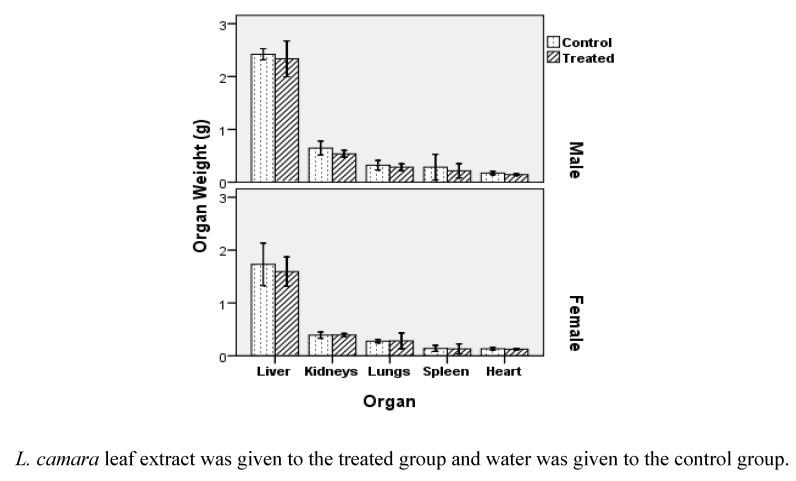
Internal organ mass of Swiss Albino mice after two weeks treatment.

Unlike body mass, male mice are more susceptible to internal organ weight alteration caused by probable *L. camara* leaf toxins. Figure 2 illustrates that the heart tissue in treated group has the most significant weight difference compared with control group, followed by kidneys (*P* = 0.031 and *P* = 0.045, respectively). It is important to note that mean body weight of males in control and treated category were nearly equal, so the effect of body mass on organ mass can be ignored in this case. The differences in body weight and organ weight may have resulted from physiological variation in rats such as food intake and metabolism. Furthermore, as mentioned, neither morbidity nor disease was observed during the entire experimentation period.

Table 1 shows the changes of biochemical parameters in the sera of male and female mice induced by *L. camara* leaf extract. In the female mice, there were slightly elevated TBIL and ALT parameters (*P* < 0.05) after exposure to *L. camara* leaf (Table 1). In the male mice, there are no significant changes for the enzymes of AST and ALP (*P* > 0.05) after oral administration of *L. camara* leaf extract. Previously, some papers reported the liver toxicity of *L. camara* leaf extract *in vivo* [14,15,16]. However, in this study only female mice showed such results which need further study. 

**Table 1:** Changes of biochemical parameters in the serum of mice induced by *L. camara*’s leaf extract.

Groups	TBIL (μmol/L)	ALT (U/L)	AST (U/L)	ALP (U/L)
**Male control**	0.9 ± 0.3	16.8 ± 3.9	78.3 ± 26.4	79.6 ± 19.9
**Male treated**	0.9 ± 0.3	17.5 ± 6.1	77.5 ± 13.4	78.6 ± 20.8
**Female control**	0.7 ± 0.3	17.1 ± 4.2	77.6 ± 12.3	76.5 ± 15.3
**Female treated**	1.0 ± 0.3 *	21.5 ± 2.5 *	76.6 ±11.6	75.9 ± 13.6

*L. camara* leaf extract was given to treated group and water was given to the control group. * *P* < 0.05.

**Figure 3:**
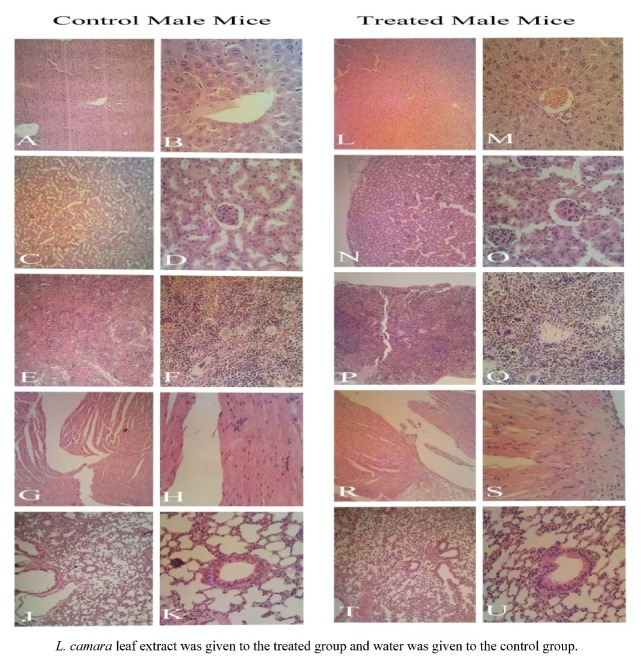
Histological (male mice) examination of A, B, L, M: Liver; C, D, N, O: Kidney; E, F, P, Q: Spleen; G, H, R, S: Heart; J, K, T, U: Lung. Magnification for left side slides of each category is 100×; right side, 400×. The focus of picture at the center in 400× magnification is on central vein in liver, glomerule in kidney, white pulp in spleen, terminal bronchiole in lung.

**Figure 4:**
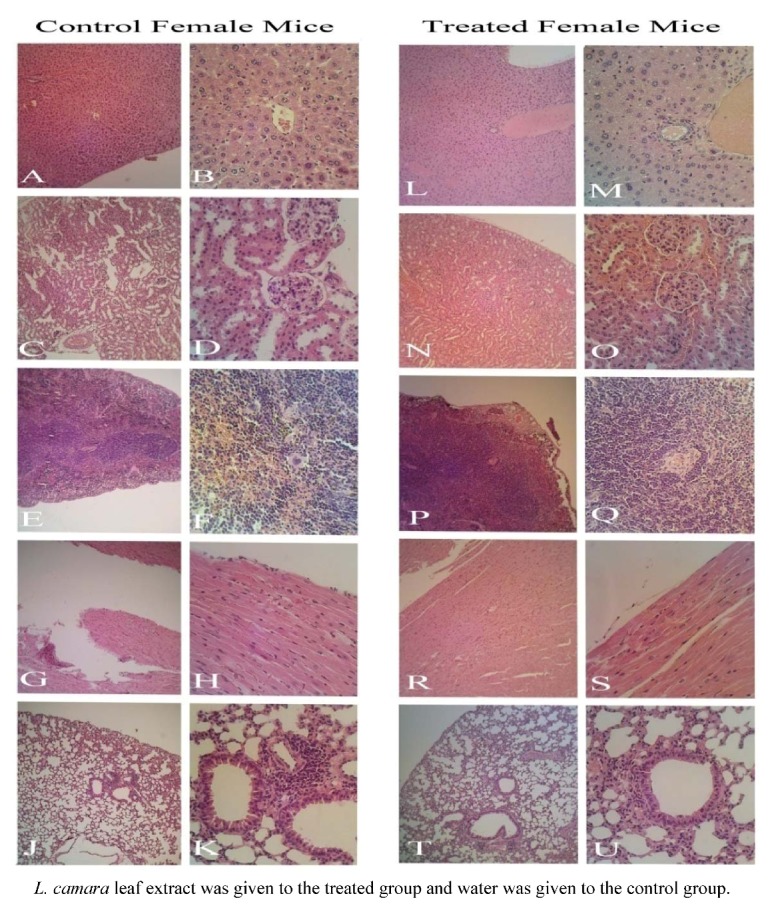
Histological (female mice) examination of A, B, L, M: Liver; C, D, N, O: Kidney; E, F, P, Q: Spleen; G, H, R, S: Heart; J, K, T, U: Lung. Magnification for left side slides of each category is 100×; right side, 400×. The focus of picture at the center in 400× powers is on central vein in liver, glomerule in kidney, white pulp in spleen, terminal bronchiole in lung.

Gross examination during autopsy and histopathological evaluations of the various organs stained with hematoxylin and eosin revealed no significant differences (Figure 3 and Figure 4). The density of Kupffer cells is apparently the same and there is no edema or hemorrhage lesion in both groups' tissues. The relative enlargement of hepatocytes' nuclei is abnormal, but common in both groups, implying the involvement of a factor other than leaf extract influence. Enlargement of hepatocytes nuclei can also be induced by physical procedure such as partial hepatectomy or the maturity of the animal which develop variation in the size hepatocytes nuclei [17].

These findings were unexpected as noticeable levels of hepatoxicity in rats caused by leaf powder of *L. camara* was reported by Saini *et al.* [18]. A Johnson and Jensen [12] study demonstrated liver toxicity in red kangaroo after ingestion of *L. camara* extract. They described liver and kidney as the most affected organs during Lantana poisoning. Furthermore, in different works, isolated compounds from *L. camara* especially lantadene A, B and C were found to produce strong hepatotoxic response in rodents [19,20,21,22]. The reason why the current study doesn’t present a strong toxicity against liver might rise from the hepatoprotective effect of some other plant constituents like oleanolic acid, as verified by Misra and Laatsch [23]. Perhaps, the mentioned compounds could have reversed the development of toxicity, healing the possible impaired tissues during the fortnight period of study. However, these need further study to verify the healing properties of *L. camara* extract.

### 2.2. Cytotoxicity Study

**Figure 5:**
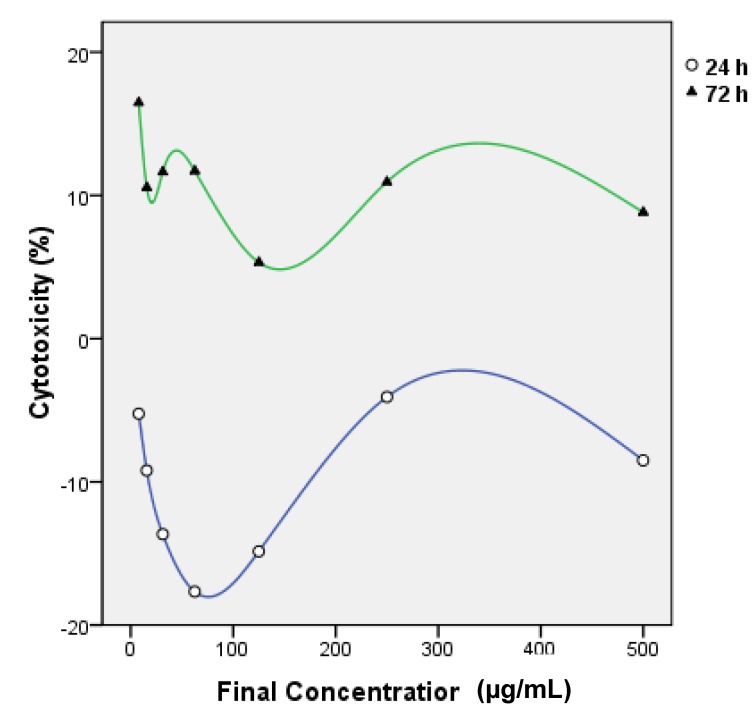
Effects of concentration and treatment time (24 h and 72 h) on cytotoxicity activity of leaf extract of *L. camara* assessed by MTT assay.

**Figure 6:**
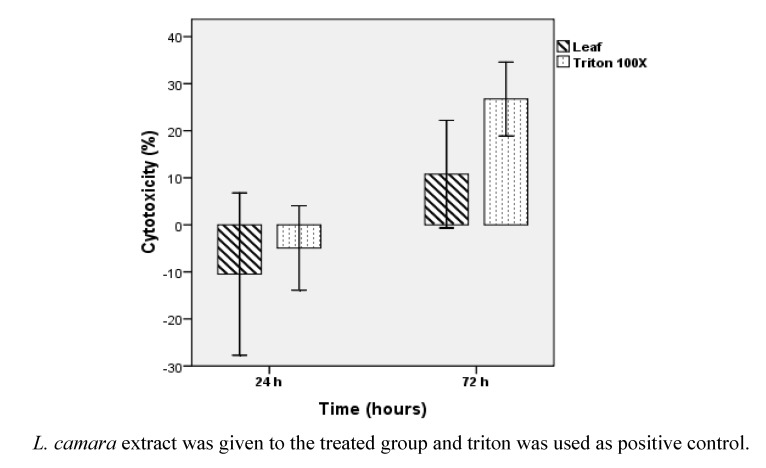
Total cytotoxicity activity of Vero cells after two different periods of treatment with leaf extract of *L. camara* and Triton 100× followed by MTT assay.

Figure 5 shows the changes in cytotoxicity of Vero cell line treated with various dilutions of *L. camara*’s leaf extract for 24 h and 72 h. IC_50 _values are summarized in Table 2. Figure 5 illustrates a higher amount of Vero toxicity after 72 h treatment, but the t test analysis doesn’t confirm a significant difference between IC_50_ mean values after 24 h and 72 h (*P* > 0.1). Hence Vero cell cytotoxicity of leaf extract is not time dependant during the period of this study. The t test results also validate that total cytotoxicity of leaf extract of *L. camara* (concentration up to 500 µg/mL) is significantly (*P* < 0.05) less than that of Triton 100× (concentration 1%) as shown in Figure 6. Vero cytotoxicity of leaf extract in maximum dilution 500 µg/mL is about 2 times and 2.5 times less than 1% Triton 100× after 24 h and 72 h treatment, correspondingly. At higher concentrations of leaf extract, the cytotoxicity decreases (Figure 5). This could suggest the existence of pro- and anti-toxic phytochemicals in the leaf extract that interfere with the cytoprotective or cytotoxic effects of each other in a concentration-dependant manner. This is the first research on Vero cytotoxicity of *L. camara*’s leaf extract.

**Table 2:** The value of IC_50_ for Vero cell line after treated with *L. camara*’s leaf extract and Triton 100×.

Sample	Time (hours)	IC_50_ (µg/mL) ± SD	
**Leaf**	24	361.44 ± 10.68	
	72	319.37 ± 99.80	
**Triton 100×**	24	-	
	72	-	

*L. camara* extract was given to the treated group and triton was used as positive control.

Recently the cell culture method was used for the production of secondary metabolites of *L. camara* cells in suspension cultures and reported to possessed cytotoxic activity [24,25,26,27]. The bioactivity of the cell-derived extract was demonstrated using cancerous HeLa cell lines. Specifically, the effect of this extract on HeLa cells was noticed from 36 h (at 100 μg/mL) to 72 h (at 25 μg/mL) by employing the 3-(4,5-dimethylthiazol-2-yl)-2,5-diphenyltetrazolium bromide (MTT) cytotoxicity assay [26]. In addition, other researchers used the aqueous extract (0.23%), obtained from callus cultures of *L. camara* (50 g dry mass), showing it had an apparent cytotoxic effect on HeLa cells with an IC_50_ value of 1,500 μg/mL in 36 h. A dose-time dependent activity of the extract was established [25].

## 3. Experimental

### 3.1. Plant Sample

*L. camara* leaves were collected from Amanjaya, Kedah, Malaysia, on February 2008. The identity of the plant was confirmed by S. Sudhakaran, in the Faculty of Applied Sciences, AIMST University, Kedah, Malaysia. A voucher with number 11008 was deposited in the herbarium of Biology School, Universiti Sains Malaysia, Penang, Malaysia.

### 3.2. Extraction Procedure

In the laboratory, the leaves sample was washed with fresh water and brushed with a soft brush before drying. Cleaned plant material was transferred to an oven (ECOCELL), for drying at 50 °C for 96 hours. Then they were powdered by electric blender. Approximately 100 g of leaf powder was added to 400 mL methanol and soaked for 4 days. Removal of leaf material from solvent was done by filtration through cheesecloth and the filtrate was concentrated using a rotary evaporator.

### 3.3. Acute Oral Toxicity Study of L. camara

#### 3.3.1. Experimental Animals

Healthy male and female Swiss albino mice (8 weeks) used for the acute oral toxicity study were bred and reared at the Animal House, Universiti Sains Malaysia, Penang, Malaysia. The animals were housed in polypropylene cages with stainless steel grill tops and provided with bedding of clean paddy husk. The animals were acclimatized to laboratory conditions for 1-week prior to treatment. The temperature in the animal room was maintained between 25 ± 2 °C with a relative humidity of 30-70% and illumination cycle set to 12 h light and 12 h dark. The mice were fed with standard laboratory pelleted feed (M/s Gold Mohur Foods and Feeds Ltd., Bangalore, India). 

#### 3.3.2. Treatment

All mice of both sexes were fasted overnight before treatment and were given food one hour after treatment. We expected that *L. camara* would be relatively safe because traditional healers use it for healing purposes. Thus, a single high dose, as recommended by Organisation for Economic Co-operation and Development [28] guidelines of 2,000 mg/kg of methanol extract dissolved in water, was administered by gavage to 10 male and 10 female mice weighing between 27 and 38 g, and water was given to 10 male and 10 female mice as a control group. After a single administration, signs of possible toxicity were observed every hour for the first six hours and every day for 14 days. Surviving animals were weighed daily and observed for any signs or symptoms of toxicity and for mortality for up to 14 days as described previously [29,30]. The visual observations included changes in the skin and fur, eyes and mucous membranes, and also in the respiratory, circulatory, autonomic and central nervous system as well as somatomotor activity and behavioural pattern. After this observation period of 14 days, seven mice of both sexes in each group were sacrificed to measure organ/body weight indices. Lung, heart, liver, spleen and kidney were removed from the remaining mice of both sexes from each group after euthanizing and killing them by cervical dislocation. This study was approved by AIMST University Animal Ethical Committee. Tissues were fixed in 10% buffered formalin. After fixation, the tissues were dehydrated in a graded series of alcohol, cleared in xylene and embedded in paraffin wax. Multiple 5 mm sections from each block were mounted on slides and stained with hematoxylin and eosin.

#### 3.3.3 Blood Biomarker Assay

In the present study, the liver function was evaluated with serum levels of total bilirubin levels (TBIL), alkaline phosphatase (ALP), alanine aminotransferase (ALT) and aspartate aminotransferase (AST).

### 3.4. Cytotoxicity Study

#### 3.4.1. Vero Cell Line

The Vero cell line was initiated from the kidney of a normal adult African green monkey on March 27th, 1962, by Yasummura and Kawakita at the Chiba University, Japan [31]. Vero cells was maintained in RPMI-1640 medium supplemented with 10% FBS, glutamine (2 mM), penicillin (100 units/mL) and streptomycin (100 μg/mL). The cells were cultured at 37 °C in a humidified 5% CO_2_ incubator.

#### 3.4.2. MTT Assay

Cytotoxicity effect of leaf extract of *L. camara* was estimated through the MTT assay described by Su *et al.* [32] with some adaptations. Vero cells were diluted with medium to 1 × 10^5^ cells/mL and aliquots (5,000 cells/50 µL) were placed in individual wells in 96-well microplate except first column wells as blank. Cells were incubated at CO_2_ 5%, 37.0 °C overnight to allow the cells to attach to the wells. Thereafter, in each well, 50 µL of leaf extract was added, which had been serially diluted 2-fold in methanol (final concentration 50% v/v), ranged from 7.8 to 500 µg/mL in final solution. Column 10 and 11 were designated as process control (Triton 100× 1%) and negative control (untreated), respectively. Next they were incubated at the above conditions for 24, 48 or 72 h and then their viability was determined by MTT color. The MTT solution (5 mg/mL in PBS, 10 µL) was added to each well and following 5 min shaking in 150 rpm, the plates were incubated for 3 h. Acidified isopropanol (100 µL) was added to each well to dissolve the formazan crystals and the plates were shaken for 20 min in 150 rpm. The absorbance of each well was read at 570 nm and at background (630 nm) on a microplate reader. Appearance of cells was monitored by inverted contrast microscope. The test was performed in triplicate. Cytotoxicity index percentage was calculated based on the following equation:

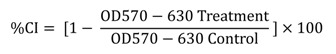

where the OD_570-630_ is absorbance at 570 nm minus absorbance at 630 nm [33]. 

#### 3.4.3. Data Analysis

Cytotoxicity percentage figures were applied to determine the mean value of IC50 (50% inhibitory concentration) by SPSS 16.00 software. T test for two independent samples was conducted to compare leaf extract and Triton 100× cell cytotoxicity. Spearman's rho nonparametric test was conducted to confirm the reduction significant in the body weight of the treated and control groups. 

## 4. Conclusions

Cytotoxicity test on Vero cell line also disclosed that leaf extract at concentrations up to 500 µg/mL inhibited the growth of cells 2.5 times less than did Triton 100× 1%. More interestingly, the cytotoxicity started to decline at elevated concentrations of this extract. While female mice lost body weight after being treated with single dose of leaf extract in acute toxicity test, male ones lost organ mass, particularly for heart and kidney. The biochemical liver function tests of serum showed significantly elevated TBIL and ALT in the *L. camara*’s leaf extract treated female mice compared with the controls (*P* < 0.05), which indicated that the liver damage was probably induced by the extract. This study presents valuable data on the acute oral toxicity of the leaf extract, which should be very useful for any future *in vivo* or clinical study of this extract. However, further toxicity studies are needed to determine the effects of this plant on chronic oral toxicity, on animal fetus, pregnant animals, and their reproductive capacity, to complete the safety profile of this extract.

## References

[1] Badakhshan M.P., Sasidharan S., Rameshwar N.J., Ramanathan S. (2009). Comparative study: Antimicrobial activity of methanol extracts of *Lantana camara*V arious Parts. Phcog. Res..

[2] Abou-Karam M., Shier W.T. (1990). A simplified plaque reduction assay for antiviral agents from plants. Demonstration of frequent occurrence of antiviral activity in higher plants. J. Nat. Prod..

[3] Afolayan A.J., Meyer J.J.M. (1997). The antimicrobial activity of 3,5,7-trihydroxyflavone isolated from the shoots of *Helichrysum aureonitens*. J. Ethnopharmacol..

[4] Hernández T., Canales M., Avila J.G., Duran A., Caballero J.R., Vivar A., Lira R. (2003). Ethnobotany and antibacterial activity of some plants used in traditional medicine of Zapotitlán de las Salinas, Puebla (México). J. Ethnopharmacol..

[5] Muthu C., Ayyanar M., Raja N., Ignacimuthu S. (2006). Medicinal plants used by traditional healers in Kancheepuram District of Tamil Nadu, India. J. Ethnobiol. Ethnomed..

[6] McKenzie R.A. (1991). Bentonite as therapy for *Lantana camara* poisoning of cattle. Vet. J..

[7] Ghisalberti E.L. (2000). *Lantana camara* L. (Verbenaceae). Fitoterapia.

[8] Mahdi-Pour B., Sasidharan S. (2011). *In vivo* toxicity study of *Lantana camara*. Asian Pac. J. Trop. Biomed..

[9] O’Neill M.J., Lewis J.A., Noble H.M., Holland S., Mansat C., Farthing J.E., Foster G., Noble D., Lane S.J., Sidebottom P.J. (1998). Isolation of translactone-containing triterpenes with thrombin inhibitory activities from the leaves of *Lantana camara*. J. Nat. Prod..

[10] Uzcategui B., Avila D., Heberto S.R., Quintero L., Ortega J., Gonzalez Y.B. (2004). Anti-inflammatory, antinociceptive and antipyretic effects of *Lantana trifolia* Linnaeus in experimental animals. Invest. Clin..

[11] Sagar L., Sehgal R., Ojha S. (2005). Evaluation of antimotility effect of *Lantana camara L*. var. acuelata constituents on neostigmine induced gastrointestinal transit in mice. BMC Complement Altern Med..

[12] Johnson J.H., Jensen J.M. (1998). Hepatotoxicity and secondary photosensitization in a red kangaroo (Megaleiarufus) due to ingestion of *Lantana camara*. J. Zoo Wildl. Med..

[13] Sharma O.P., Dawra R.K., Makkar H.P. (1988). Effect of polymorphic crystal forms of lantana toxins on icterogenic action in guinea pigs. Toxicol. Lett..

[14] Garg S.K., Shah M.A., Garg K.M., Farooqui M.M., Sabir M. (1997). Antilymphocytic and immunosuppressive effects of *L. camara* leaves in rats. Indian J. Exp. Biol..

[15] Black H., Carter R.G. (1985). Lantana poisoning of cattle and sheep in New Zealand. N. Z. Vet. J..

[16] Sharma O.P., Dawra R.K., Makkhar H.P. (1989). Toxicity of isolated lantana (*L. camara* L.) constituents to male and female guinea pigs. Vet. Hum. Toxicol..

[17] Jackson M.R. (1974). The nature of dimethylnitrosamine induced enlargement of rat hepatocyte nuclei. J. Pathol..

[18] Saini N., Singh J., Sehgal R., Ojha S. (2007). Evaluation of liver function impairment and lipid peroxidation induced by *Lantana camara* leaf powder administration in adult rat serum and liver. Cell Mol. Biol. (Noisy-le-grand).

[19] Sharma O.P., Dawra R.K., Makkar H.P. (1987). Isolation and partial purification of Lantana (*Lantana camara* L.) toxins. Toxicol. Lett..

[20] Sharma O.P., Vaid J., Pattabhi V., Bhutani K.K. (1992). Biological action of lantadene C, a new hepatotoxicant from *Lantana camara* var. aculeata. J. Biochem. Toxicol..

[21] Hart N.K., Lamberton J.A., Sioumis A.A., Suares H., Seawright A.A. (1976). Triterpenes of toxic and non-toxic taxa of *Lantana camara*. Experientia..

[22] Zheng H.Q., Wei N., Wang L.F., He P. (2006). Effects of *Lantana camara* leaf extract on the activity of superoxide dismutase and accumulation of H_2_O_2_ in water hyacinth leaf. ZhiWu Sheng Li Yu Fen Zi Sheng Wu Xue Xue Bao..

[23] Misra L., Laatsch H. (2000). Triterpenoids, essential oil and photo-oxidative 28 --> 13-lactonization of oleanolic acid from *Lantana camara*. Phytochemistry.

[24] Srivastava P., Sisodia V., Chaturvedi R. (2011). Effect of culture conditions on synthesis of triterpenoids in suspension cultures of *Lantana camara* L. Bioprocess Biosyst. Eng..

[25] Srivastava P., Kasoju N., Bora U., Chaturvedi R. (2009). Dedifferentiation of leaf explants and cytotoxic activity of an aqueous extract of cell cultures of *Lantana camara* L. Plant Cell Tissue Organ Cult..

[26] Srivastava P., Kasoju N., Bora U., Chaturvedi R. (2010). Accumulation of Betulinic, Oleanolic, and Ursolic acids in in vitro cell cultures of *Lantana camara* L. and their significant cytotoxic effects on HeLa cell lines. Biotechnol. Bioprocess Eng..

[27] Srivastava P., Chaturvedi R. (2010). Simultaneous determination and quantification of three pentacyclic triterpenoids-betulinic acid, oleanolic acid, and ursolic acid-in cell cultures of *Lantana camara* L. 2010. In Vitro Cell Dev. Biol. - Plant.

[28] (1992). OECD Guidelines for Testing of Chemicals. No 420: Acute Oral Toxicity-fixed Dose Method.

[29] Lee J.N., Park C.S., Kim H.P., Hwang S.Y., Chung W.G. (2002). Single dose toxicity study of Hwangjaegongjinbo, an invigorator, in mice and rats. J. Toxicol. Pub. Health.

[30] Ryu S.D., Park C.S., Baek H.M., Baek S.H., Hwang S.Y., Chung W.G. (2004). Antidiarrheal and spasmolytic activities and acute toxicity study of Soonkijangquebo, a herbal anti-diarrheal formula. J. Ethnopharmacol..

